# Datasets for multi-scale diffraction analysis (synchrotron XRD and EBSD) of twinning-detwinning during tensile-compressive deformation of AZ31B magnesium alloy samples

**DOI:** 10.1016/j.dib.2019.104423

**Published:** 2019-08-28

**Authors:** Hongjia Zhang, Antoine Jérusalem, Enrico Salvati, Chrysanthi Papadaki, Kai Soon Fong, Xu Song, Alexander M. Korsunsky

**Affiliations:** a Department of Engineering Science, University of Oxford, Oxford, UK; b Singapore Institute of Manufacturing Technology, Singapore

**Keywords:** Twinning-detwinning, Crystal plasticity, EBSD, Synchrotron XRD

## Abstract

Diffraction data were collected using synchrotron X-ray scattering (sXRD) and electron back-scattered diffraction (EBSD) during *in situ* tensile-compressive deformation of Mg alloy AZ31B dogbone samples. The onset and evolution of twinning and detwinning were monitored based on intensity changes in sXRD 2D scattering patterns (which also provided average elastic strain values through the calculation of orientation-specific lattice spacing changes), and EBSD, that revealed the micro-scale grain morphology changes. The observations were interpreted and analysed with the help of crystal plasticity finite element modelling (CP-FEM), as reported in the published article (https://doi.org/10.1016/j.ijplas.2019.02.018).

**Table:** Specifications Table

Subject	Materials Science and Engineering
Specific subject area	Structure and mechanical properties
Type of data	Synchrotron X-ray diffraction 2D detector patterns EBSD grain orientation maps Mechanical testing (tension-compression) data Code for data analysis and plotting
How data were acquired	Simulation: ABAQUS SEM: TESCAN LYRA3 FIB-SEM EBSD: NordlysNano, Oxford Instruments Deben Loading Rig: 5kN MT10223 from DEBEN Synchrotron X-Ray diffraction: Beamline B16, Diamond Light Source Photonic Science ImageStar 2D detector
Data format	Raw: TIFF, XLS Analysed Filtered
Parameters for data collection	Dogbone samples of magnesium alloy AZ31B were loaded uniaxially using DEBEN MicroTest rig at the rate of 0.2mm/min to −250 N (max compressive stress −112MPa), followed by tensile loading to fracture at ∼560 N. The compression-tension loading was also perfumed *in situ* during X-Ray diffraction with 18keV monochromatic X-Ray beam (size 1 mm × 1 mm). I*n situ* EBSD was also carried out within SEM at 25kV accelerating voltage and spatial resolution 0.8 μm. Four EBSD maps of the same ROI within the sample were collected during loading to observe twinning-detwinning processes in HCP polycrystal Mg alloy.
Description of data collection	Photonic Science ImageStar 9000 detector (3056 × 3056 matrix, 31 μm pixel size) was placed ∼130mm from the sample to increase angular resolution for strain evaluation. Exposure time was set to 200s to allow peak fitting and prevent pixel saturation for three Debye-Scherrer rings seen in the diffraction patterns. Load control mode was chosen during exposure to maintain uniform elastic lattice strain. For EBSD, four markers were deposited using Pt GIS at four map corners to ensure consistent map position under *in situ*. Load cell signal was maintained constant over several hours to perform EBSD mapping. Data were post-processed by CHANNEL 5 software and MTEX Matlab package.
Data source location	University of Oxford, Oxford, United Kingdom
Data accessibility	The data is stored in accordance with Open Access arrangements on Mendeley Data and can be downloaded following the links below: Repository name: Mendeley Data Data identification number: https://doi.org/10.17632/4c376dt9kw. Direct URL to data: https://data.mendeley.com/datasets/4c376dt9kw/2
Related research article	Author's name: Hongjia Zhang, Antoine Jérusalem, Enrico Salvati, Chrysanthi Papadaki, Kai Soon Fong, Xu Song, Alexander M. Korsunsky Title: Multi-scale mechanisms of twinning-detwinning in magnesium alloy AZ31B simulated by crystal plasticity modelling and validated via *in situ* synchrotron XRD and *in situ* SEM-EBSD Journal: International Journal of Plasticity DOI: https://doi.org/10.1016/j.ijplas.2019.02.018

**Table:** 

**Value of the data** • This comprehensive set of experimental measurements provides unique correlated data from X-ray and electron diffraction measurements of Mg alloy AZ31B sample(s) subjected to reversed compression-tension deformation. The data can be used to validate and calibrate future numerical models against spatially (EBSD) and orientationally (sXRD) micro-resolved measurements. • The data will benefit experimentalists and modellers exploring deformation mechanisms and their interaction (particularly slip vs twinning-detwinning) in metals with HCP structure, e.g. alloys of Mg, Ti, Zr • Data provides insight into the conditions for the onset of twinning-detwinning under external loading, and will be used to plan and design further experiments with better confidence • The multi-scale nature (Type I, II and III) of the experimental and numerical data provides uniquely complete description of the twinning-detwinning process in Mg alloys

## 1 Data

The data provided in this paper relate to the paper published in the International Journal of Plasticity [1]. With reference to the Mendeley Data dataset, all data files are described individually below.

**Figure:**
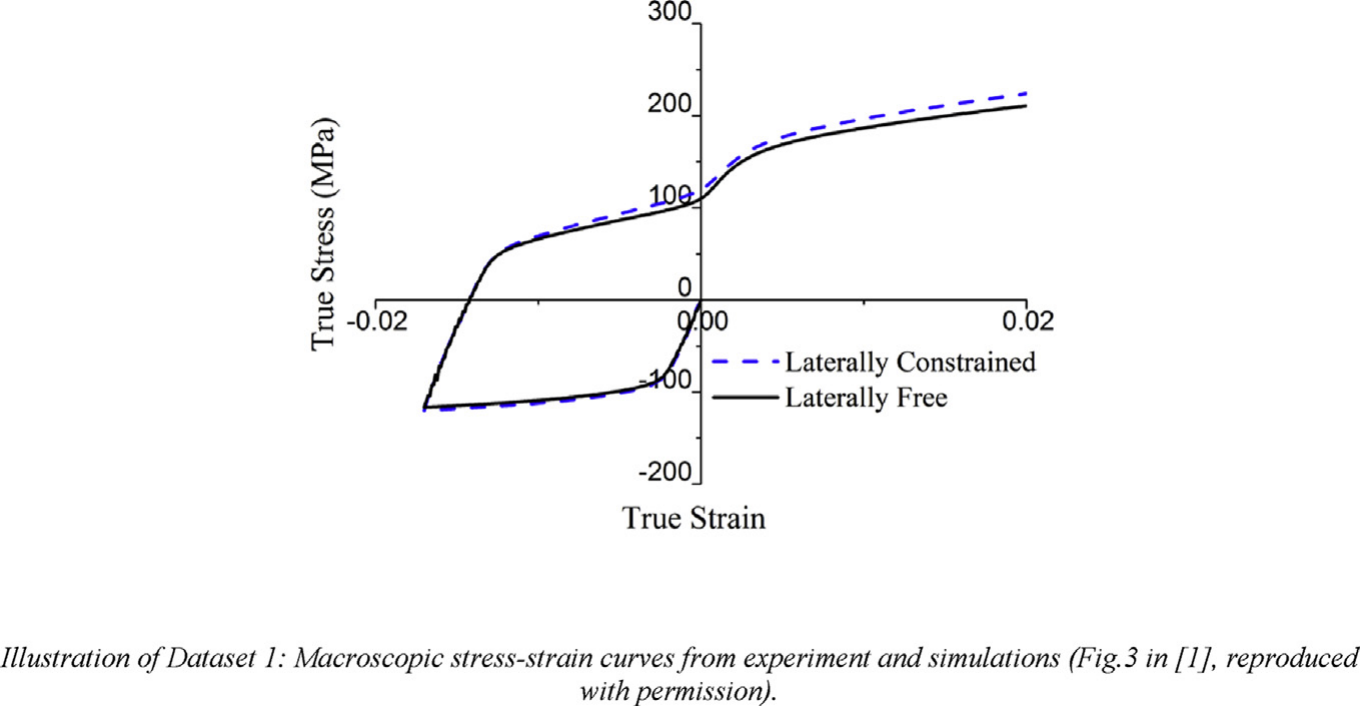

Dataset1 (referring to Figure 3 in Ref. [1]): Stress-strain curves extracted from the CPFEM simulations with the same parameters except for boundary conditions which are respectively laterally constrained and laterally free for the surfaces perpendicular to the loading axis, in order to examine the effects that boundary conditions have on the simulation results.

**Figure:**
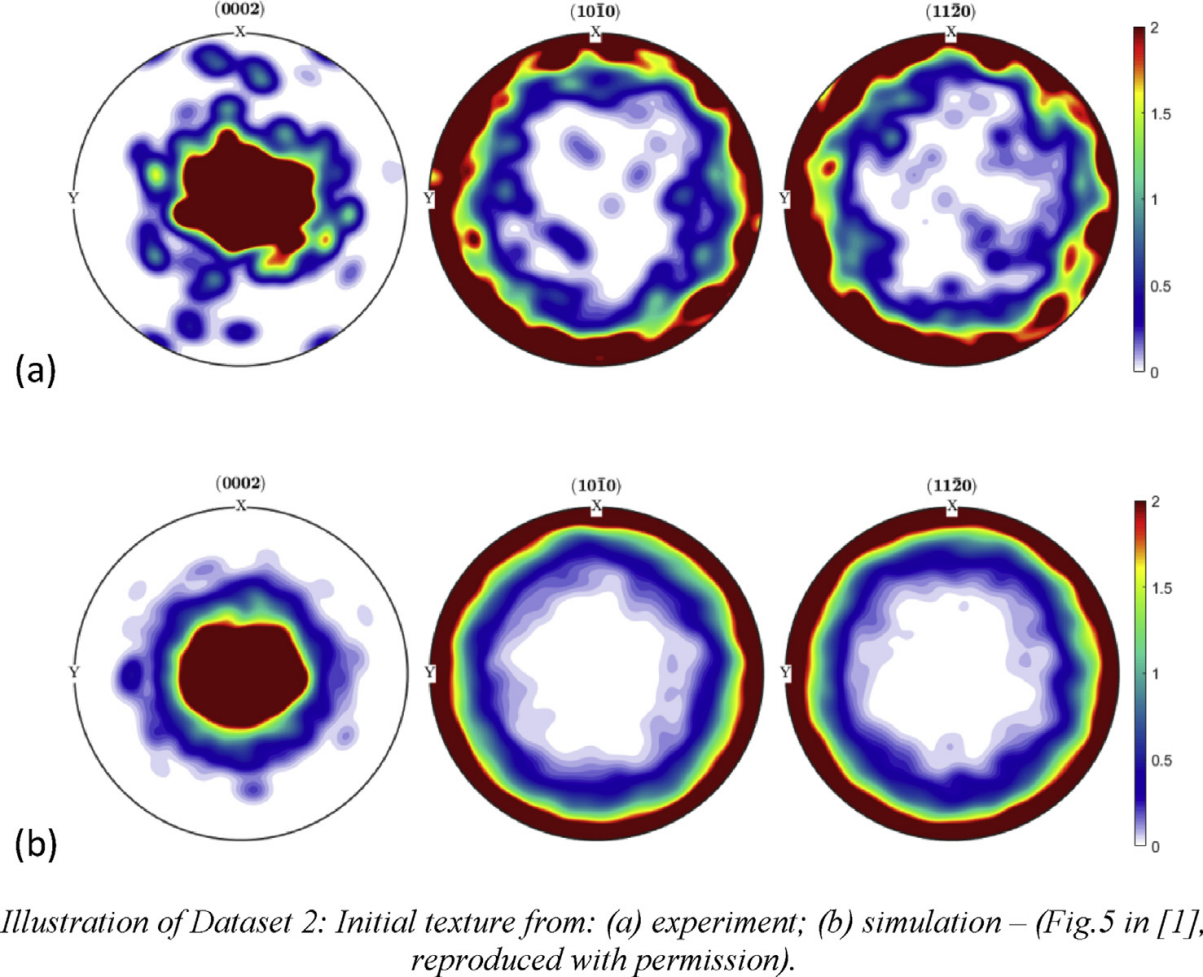

Dataset2 (referring to Figure 5 in Ref. [1]): Experimental and numerical data of initial Euler angles (ZXZ convention) of grains in the polycrystal magnesium alloy obtained via EBSD and CPFEM simulation. Normalised pole figures of initial texture are drawn using these angles with MTEX in Matlab.

**Figure:**
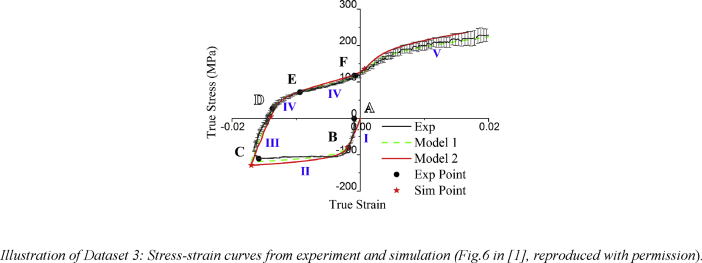

Dataset3 (referring to Figure 6 in Ref. [1]): Stress-strain curves related to macroscopic compression-tension experiment and CPFEM simulations with Model 1 (one-grain-one-element) and Model 2 (one-grain-multiple-element), in order the compare simulation with experiment.

**Figure:**
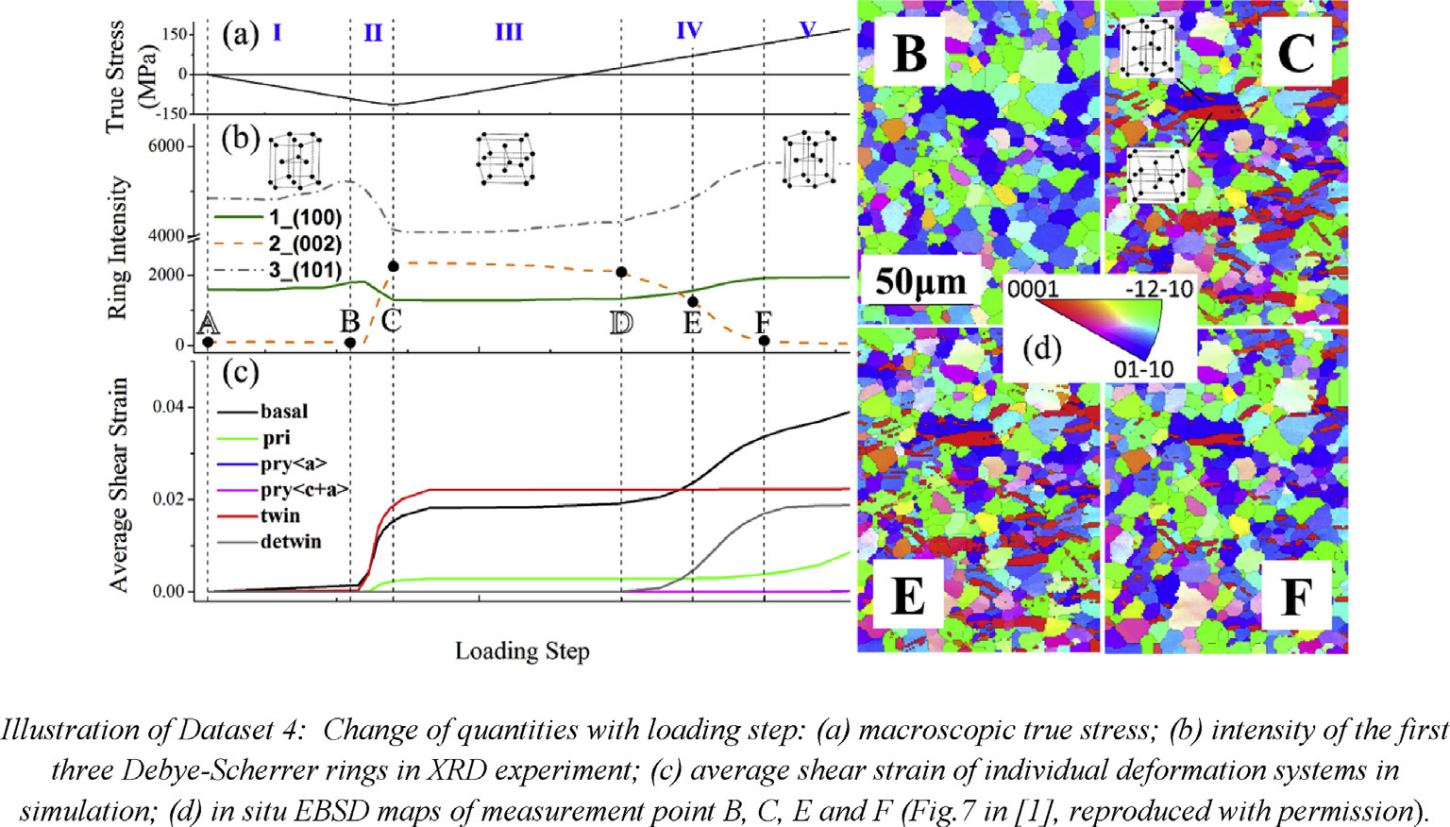

Dataset4 (referring to Figure 7 in Ref. [1]): Experimental data in curves showing the change of the intensity of three diffraction rings corresponding to different crystal planes, which reveals the beginning and ending of twinning detwinning. Curves obtained numerically demonstrating the amount of shear strain caused by various deformation systems including basal slip, prismatic slip, pyramidal <a> slip, pyramidal <c+a> slip, twinning and detwinning. All above are correlated with macroscopic loading stress.

**Figure:**
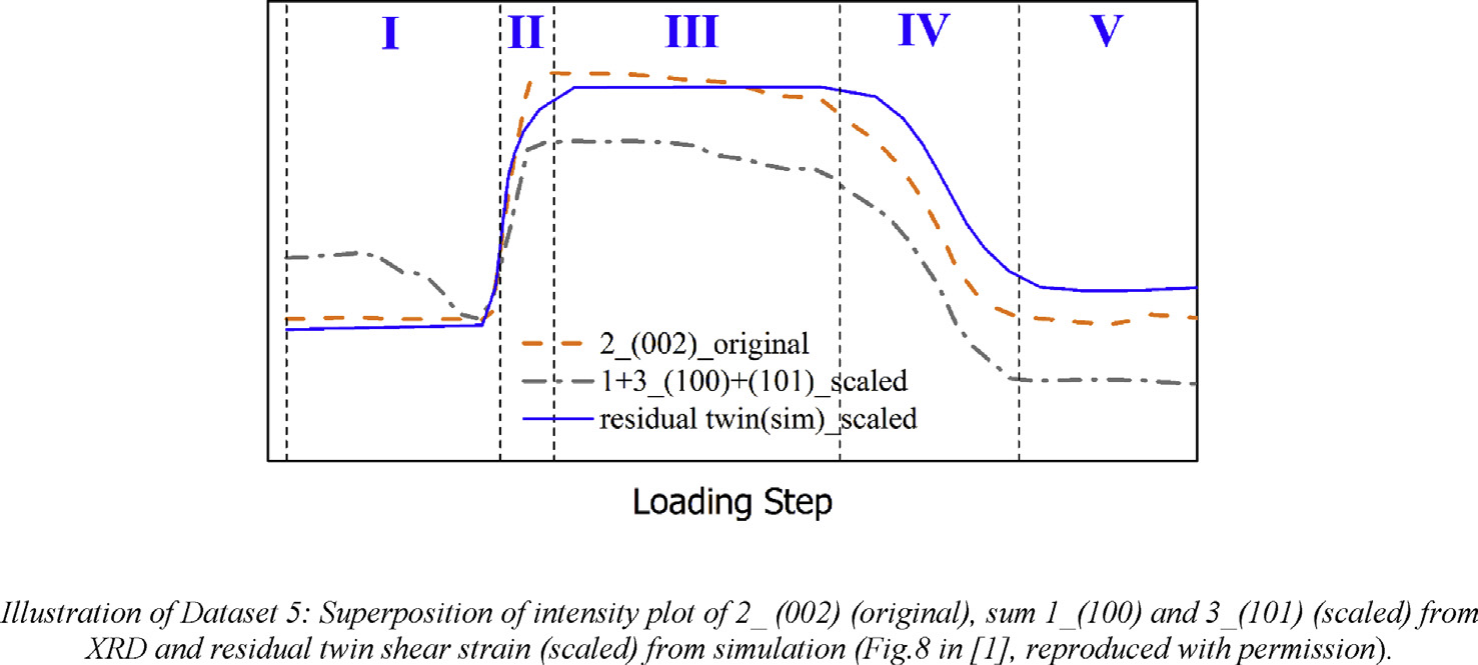

Dataset5 (referring to Figure 8 in Ref. [1]): Curves of diffraction ring intensity change obtained from *in situ* XRD experiment for the 2nd ring (original) and the sum of the 1st and 3rd rings (scaled) with plot of residual twin shear strain (scaled) from CPFEM simulation.

Dataset6-1: Raw diffraction patterns collected during *in situ* XRD experiment with macroscopic compression-tension loading. The accompanying excel file (named Dataset6-2) indicates the loading (stress) applied for each of the diffraction patterns.

## 2 Experimental design, materials, and methods

Data from various experiments and CPFEM simulation on commercially available wrought magnesium alloy AZ31B are provided in this paper. The average grain size of the material was roughly 20 μm with typical rod texture after manufacturing processes.

For experiments, macroscopic compression-tension loading test was performed on dog-bone shaped samples to separately, with *in situ* EBSD and with *in situ* XRD. Constant loading rate of 0.2mm/min was applied by Deben (5kN, MT10223) throughout the loading processes. The engineering compressive stress of −127 MPa (−250 N) was reached at maximum before reverse loading till fracture at nearly 284 MPa (560 N) engineering tensile stress. True stress and true strain are calculated according to the gripping length of the sample and are provided in the data file.

A total of 57 diffraction patterns were collected during high energy XRD experiments with *in situ* compression-tension loading (mentioned above) performed at Beamline B16, Diamond Light Source. The x-ray beam was monochromatic with 18 keV energy and shaped to the size of 1 mm × 1 mm with slits. The sample was placed 130mm away from the detector (Photonic Science X-Ray Image Star 9000) with resolution of 3056 × 3056pixels and pixel size of 31 μm. The sample-detector distance made sure that only the first three diffraction rings could appear on the diffraction patterns. The detector was exposed 200 s for the collection of each diffraction pattern. Raw diffraction patterns are provided in the zip file with an excel sheet explaining the loading condition under which the diffraction pattern was taken. To obtain the intensity change of each diffraction ring, the conventional ‘caking’ in Fit2D [2] of diffraction rings was performed, which was basically radial binning with azimuthal integral. The angle of azimuthal integral was set to be from 75° to 105° (0° set to face ‘east’ on the pattern). The macro script used in Fit2D for ‘caking’ is also provided named as ‘Program1_caking.mac’.

*In situ* EBSD loading was performed inside the chamber of the SEM. The compression-tension loading was the same as descripted above. After careful mechanical polish, the sample was placed on a pre-designed 70° tilted grip to satisfy the geometrical requirement of EBSD experiment. During the loading process, EBSD mapping (25kV and 0.8 μm spatial resolution) of a rectangle area on the sample surface was carried out four times with the loading cell being held at a fixed position. To make sure the EBSD detector was mapping the same sample surface area, four markers were deposited with Pt sputtering at the corners of the rectangle area. At what loading stress to perform the EBSD mapping was decided beforehand with the information from XRD experiments so that the four maps correspond to the stage right before the inception of twinning, at the maximum compressive stress (with the largest amount of twinning strain), in the middle and at the end of detwinning process, respectively. Software Tango from CHANNEL 5 system (Oxford Instruments) was used to perform noise reduction on the raw data (mainly Euler angles) from EBSD mapping and the resulted data are provided here.

In total, 2592 grains were included in the CPFEM simulation model. The grain orientations represented with Euler angles were generated with 3D Voronoï tessellations and assigned to 2592 elements in Model 1 (one-grain-one-element) and 475,947 elements in Model 2 (one-grain-multiple-element). State variables in the VUMAT subroutine in ABAQUS were set to represent the amount of shear strain caused by multiple deformation systems and were used to draw Fig. 7 and Fig. 8.

MTEX in Matlab was used to plot normalised pole figures using Euler angles from EBSD and CPFEM simulation. MTEX script ‘Program2_pole-figure-plot_normalised.m’ is included in the Mendeley Dataset.
